# Morphological study of safe fixation region of temporomandibular joint prosthesis in Chinese northeast population with 3-dimensional computed tomographic image

**DOI:** 10.1097/MD.0000000000022779

**Published:** 2020-10-23

**Authors:** Zhuan Zhong, Jialiang Sun, Zhentao Yu, Yingying Han, Chunyang Kang

**Affiliations:** a Department of Neurology, China-Japan Union Hospital of Jilin University; b Department of Orthopaedics, The Second Hospital of Jilin University; c Department of Pediatrics, The First Hospital of Jilin University; d Department of Gastrointestinal Colorectal and Anal Surgery, China-Japan Union Hospital of Jilin University, 130033 Changchun, Jilin, China.

**Keywords:** Chinese, computed tomography, measurement, prosthesis, temporomandibular joint

## Abstract

This study aimed to measure temporomandibular joint (TMJ) with 3-dimensional (3D) reconstruction technique in Chinese northeast population, and to clarify the region for fixation and to provide morphological basis for the application of TMJ prosthesis in Chinese setting.

Computed tomography (CT) scan and 3D reconstruction were performed with 132 individuals. Structural markers and measurements were further performed with a 3D model of the total TMJ, including the width, thickness and angle of zygomatic arch, the width and height of articular fossa, as well as the area, width, thickness and angle of mandible in the fixation region of the TMJ prosthesis. All the measured indicators values were compared between bilateral sides and gender groups.

There was no statistical difference in the measured indicators between the left side and the right side (*P* > .05). However, certain parameters, including S, L5, L7, P4, and P5, were significantly different among males and females (*P* < .05).

In this study, 3D CT image was used to obtain the measurement data of TMJ, which provided data support for the clinical application of TMJ prosthesis in Chinese population.

## 1 Introduction

Temporomandibular joint (TMJ) is composed of the articular fossa of temporal squama, the mandibular condyle, and the articular disc, as well as the surrounding ligaments between them. A variety of TMJ diseases can cause structural damage and then affect relevant physiological functions, thus further reducing the quality of life of the patients.^[1]^ In clinical practice, surgical repairing and functional reconstruction are always used to treat the diseases. TMJ prosthesis is one of the most important therapeutic methods, including TMJ Concepts/Techmedica personalized prosthesis and standard Biomet/Lorenz TMJ prosthesis, which are well accepted by both physicians and patients worldwide.^[2,3]^ However, there have been studies indicating that these prostheses may not be suitable in Chinese setting due to the differences in the morphological parameters of TMJ and the design of the prosthesis of Chinese population compared with the western countries.^[4,5]^ At present, there have been certain studies reporting preliminary findings with respect to the application of TMJ prosthesis in China.^[6,7]^ In this study, we aimed to perform a detailed and in-depth analysis of the fixed region of the prosthetic screw by measuring the zygomatic arch, condyle, and mandibular data in Chinese northeast population, so as to provide morphological data for the application of TMJ prosthesis.

## 2 Materials and methods

**Figure 1:**
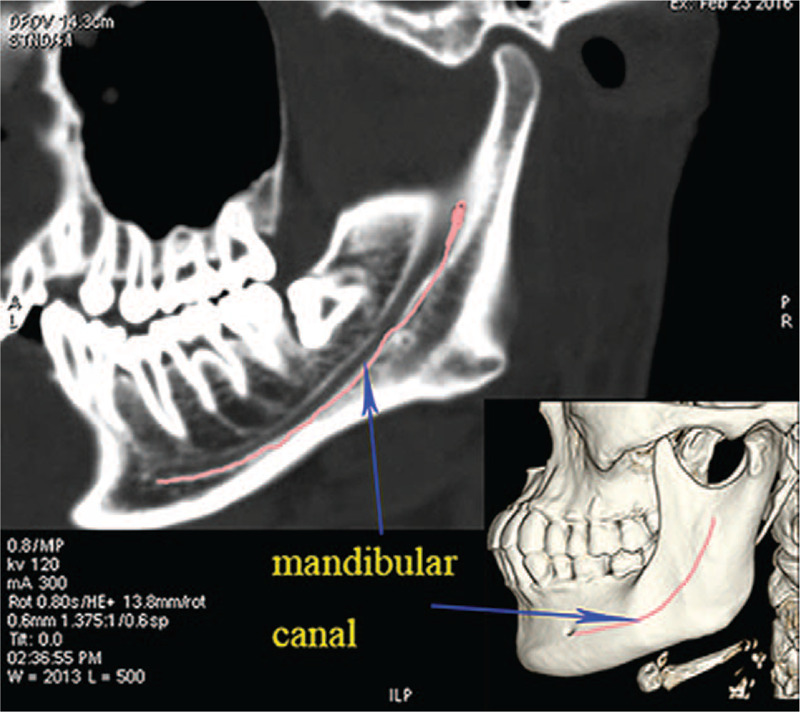
Real-time mark of the mandibular canal in the reconstructed CT image and the 3D skull model.

The data of whole-brain computed tomography (CT) scans of 132 volunteers from February 2015 to December 2017 were obtained from the China-Japan Union Hospital of Jilin University in accordance with ethical committee guidelines. Among the incorporated 132 cases, 131 cases were Chinese Hans and 1 case was Chinese Hui. And there were 65 cases of males and 67 cases of females, ranging from 22 to 65 years old. All the selected patients had natural dentition, without anodontism, previous histories of TMJ disorders, maxillofacial trauma or maxillofacial deformity, while the bone structures of bilateral TMJ were not affected. Bilateral data were measured in all patients with mouth closed. All CT images were obtained by Siemens 64-row spiral CT scanner. The reconstruction system (GE ADW 4.5; GE) was used for skull reconstruction. The images were displayed simultaneously in 3 orthogonal planes (axial, sagittal, and coronal). After skull reconstruction, the parameters could be measured at any desired plane, and the mandibular canal could be clearly revealed and be marked in real time as well, as shown in Figure 1.

**Figure 2:**
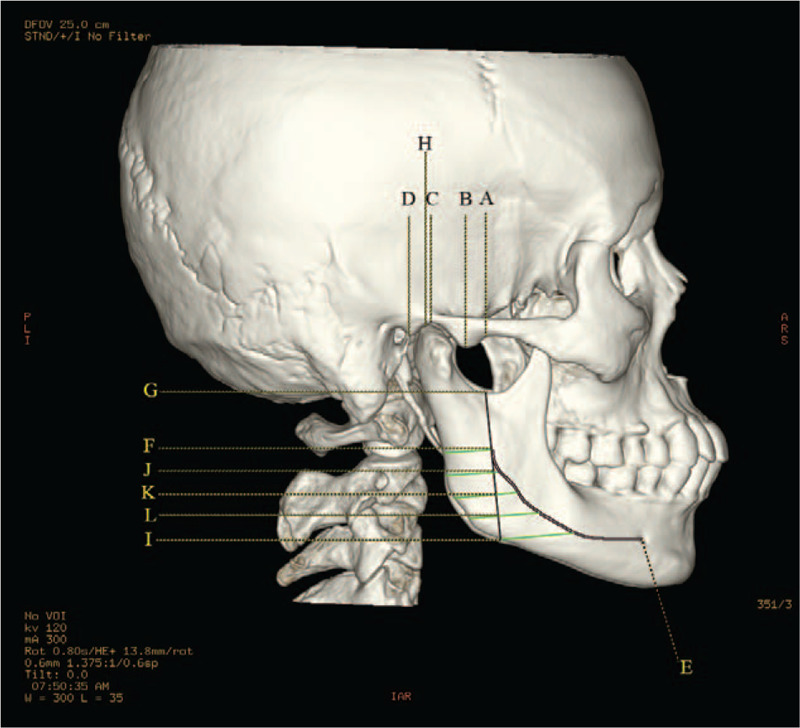
Measurement points on the zygomatic arch, articular fossa, and mandibular ramus on the 3-dimensional skull.

**Figure 3:**
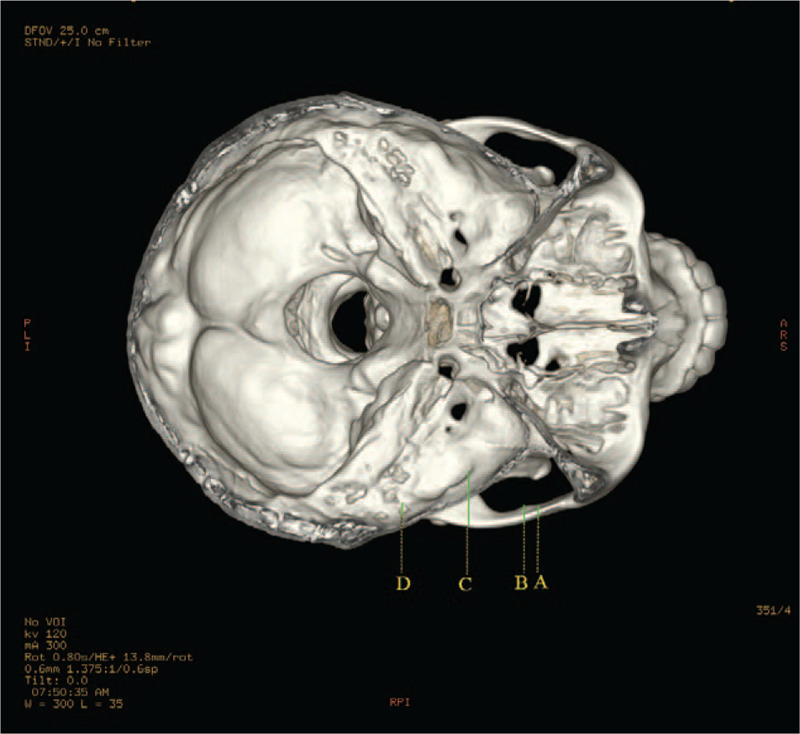
Measurement of bone thickness marked as green on the selected point of zygomatic arch.

The determination and measurement of the landmarks of the zygomatic arch were as follows. First of all, the starting point of articular tubercle was defined as point A, and the lowest point of articular tubercle was named as point B, while the highest point of the lateral margin of the articular fossa was point C, and the lowest point of the posterior articular process was determined as point D. Then, 4 straight lines (L1, L2, L3, and L4) perpendicular to the upper edge of the zygomatic arches from point A, B, C, and D were formed, the length of which was defined as the height of the zygomatic arch at the 4 points (A, B, C, and D). Afterwards, the midpoints of the 4 line segments were marked as point a, b, c, and d, respectively, where the thicknesses of the bone were measured and recorded as D1, D2, D3, and D4. Finally, the lengths between the 4 midpoints (line segments of ab, bc, cd, and ad) were expressed measured, as shown in Figure 2. The angles of ∠abc (α) and ∠bcd (β) were also measured along the OML plane, as shown in Figure 3.

**Figure 4:**
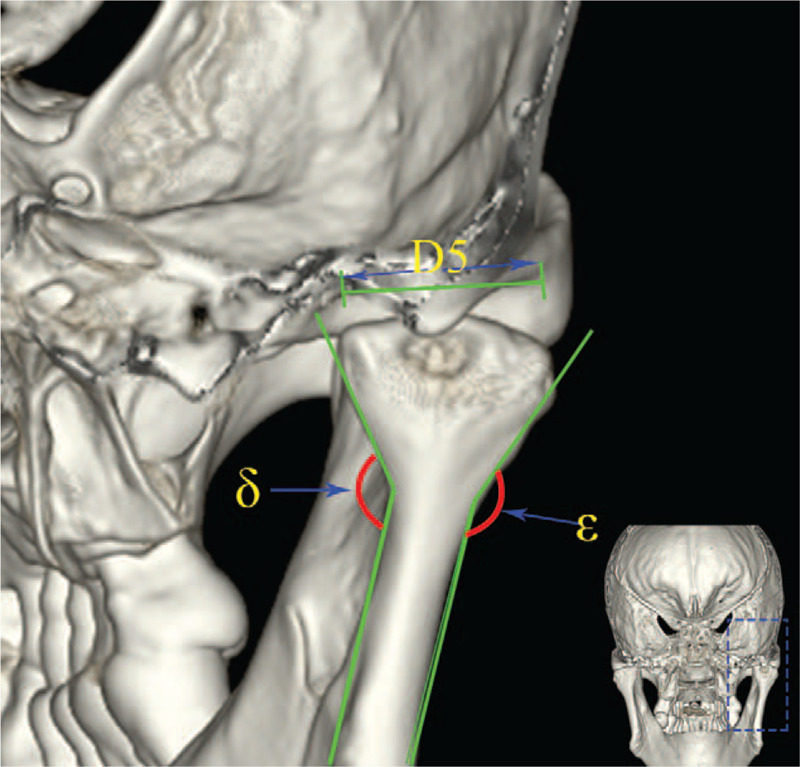
Measurement of interior inclination angle and exterior inclination angle of the processus condylaris and the transversal diameter of articular fossa.

The landmarks of the articular fossa were determined as follows. On the basis of previous definition, the length of the line segment between point B and D was marked as BD. Then, a line perpendicular to the BD line segment was constructed from point C, the length of which was marked as H1. Meanwhile, the distance between the lateral point and the medial point of the articular fossa was measured and marked as D5, as shown in Figure 4.

The landmarks of mandibular ramus were determined and measured as follows. The lowest point of foramina mentale was labeled as point E, the mandibular foramina as F, the lowest point of the mandibular notch as G, and the highest point of the condyle as H. The intersection point of the mandible and the extended FG line segment was marked as point I. A line perpendicular to FG line segment was constructed from point G, and the distance between point H and the perpendicular line was marked as L5. The lengths of line segments FG and FI were defined as L6 and L7, respectively. The angle between the inferior radiographic base line and FG line segment was marked as γ. The maximum curvature of the mandibular ramus was considered as the apex, and interior inclination angle ε and exterior inclination angle δ of the processus condylaris were further measured. The FI line segment was divided into 4 sections with equal length, and the demarcation points were named as point J, K, L, respectively, from the top to bottom. A line segment perpendicular to FG line segment was constructed. The intersection of the 5 lines and the projection of the mandibular canal on the mandibular branch and mandibular ramus were taken as the starting and ending points to form 5 line segments for the measurement of the area (S) around the mandibular ramus. The lengths of 5 line segments were measured from top to bottom and labeled as P1, P2, P3, P4, and P5. The bone thicknesses were measured at the midpoint of 5 lines and were as labeled as M1, M2, M3, M4, and M5 successively from top to bottom, as shown in Figure 1.

All statistical analyses were performed with SPSS (version 19.0; SPSS Inc, IBM, Chicago, IL). The normality test of the parameters in this study showed that they conformed to the normal distribution. Therefore, the measurements were presented as mean ± standard deviation (SD), including L1, L2, L3, L4, D1, D2, D3, D4, ab, bc, cd, ad, α, β, BD, H1, D5, S, L5, L6, L7, P1, P2, P3, P4, P5, M1, M2, M3, M4, M5, γ, ε, and δ. Independent-sample *t* test was performed to compare the mean differences between groups, and 1-way analysis of variance (ANOVA) was applied for the comparison of parameters among zygomatic arch and mandibular ramus, with an inspection level of α = 0.05. *P* value less than .05 considered as the criteria for statistical significance.

## 3 Results

**Figure 5:**
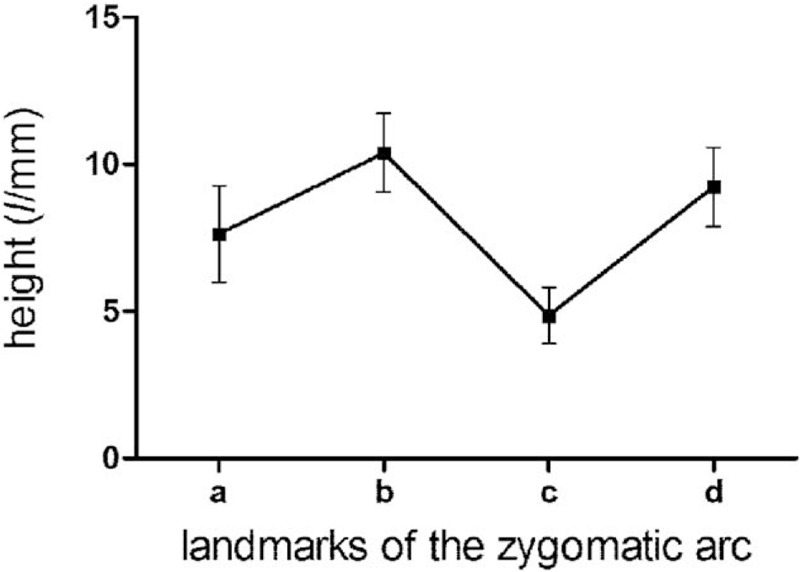
Changes of height of the zygomatic arch at different landmarks.

**Figure 6:**
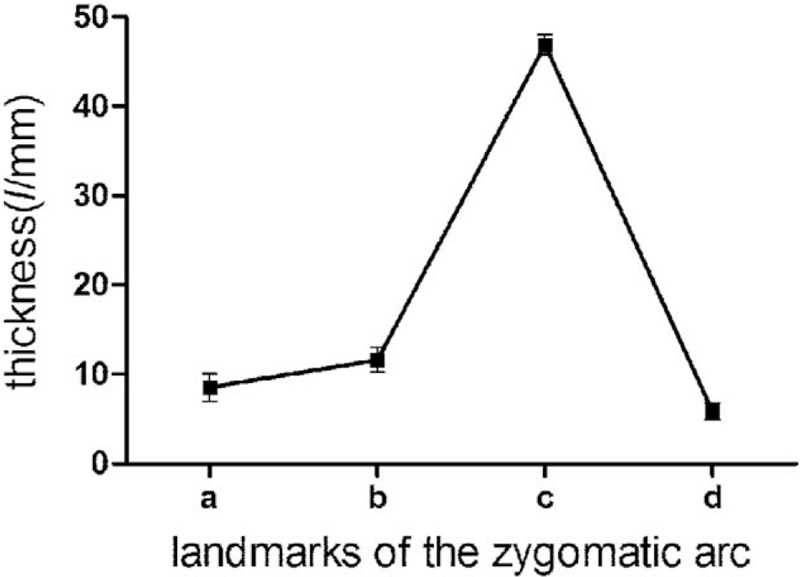
Changes of thickness of the zygomatic arch at different landmarks.

The measurement results of indexes (L1, L2, L3, L4, D1, D2, D3, D4, ab, bc, cd, ad, α, β) of the zygomatic arch of 2 sides are summarized in Table 1, for which no significant difference was observed between the 2 sides (*P* > .05). Moreover, there is no statistically significant difference between males and females either (*P* > .05), as summarized in Table 2. The ANOVA results indicated that the heights of the zygomatic arch at the 4 points were not completely equal (L1, L2, L3, and L4) (*P* < .001), The height of L3 was the shortest, while the height of L2 was the longest among the 4 heights (*P*_L3vsL2_ < .001, *P*_L3vsL4_ < .001, *P*_L3vsL1_ < .001, *P*_L2vsL4_ < .001). The changes of heights and thicknesses of the zygomatic arch are shown in Figures 5 and 6.

**Table 1:**
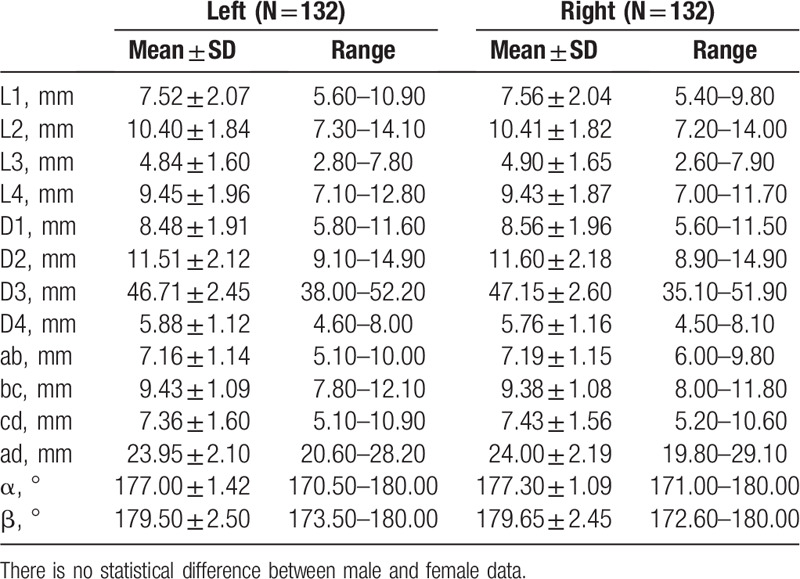
Width, thickness, and angle of the measuring points on zygomatic arc between 2 sides.

**Table 2:**
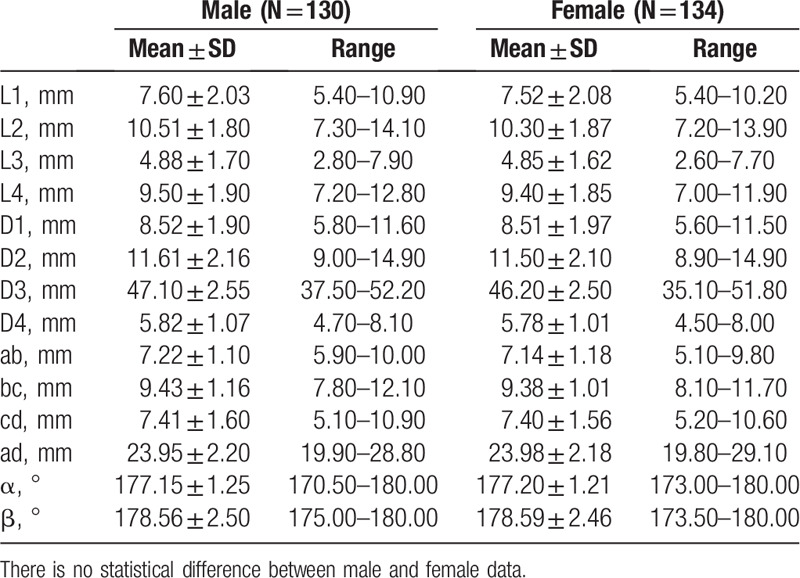
Width, thickness, and angle of the measuring points on zygomatic arc between 2 genders.

As summarized in Table 3, no statistical difference was observed in the indexes of the articular fossa (BD, H1, and D5) between 2 sides (*P* > .05). Similarly, no statistically significant difference was discovered among males and females either (*P* > .05), as summarized in Table 4.

**Table 3:**
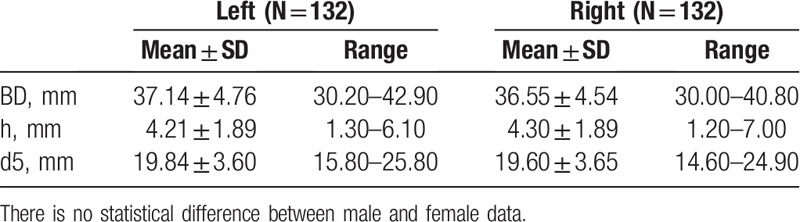
Width and height of the measurement points on articular fossa between 2 sides.

**Table 4:**
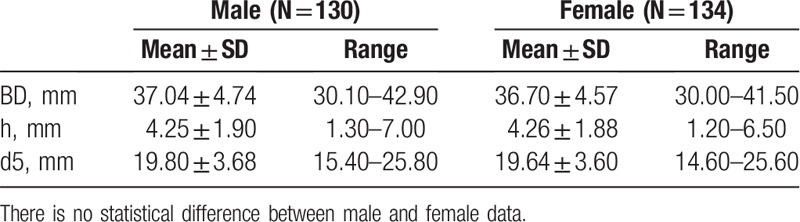
Width and height of the measurement points on articular fossa between 2 genders.

**Figure 7:**
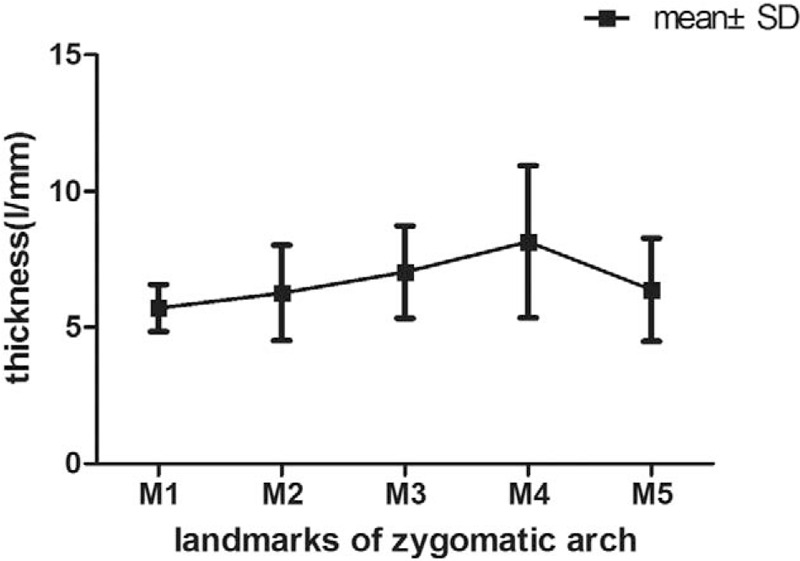
Changes of thickness of the mandibular ramus at different landmarks.

Table 5 presented that no significant difference was discovered in all the indexes of the mandibular ramus (S, L5, L6, L7, P1, P2, P3, P4, P5, M1, M2, M3, M4, M5, γ, ε, and δ) between 2 sides (*P* > .05). It was suggested that the significant differences between males and females were only found in indexes of S (*P* < .001), L5 (*P* = .028), L7 (*P* = .019), P4 (*P* = .023), and P5 (*P* = .027) (*P* < .05), as summarized in Table 6. The ANOVA analysis indicated significant increased lengths from the projection of the mandibular canal to the posterior edge of the mandibular ramus (P1, P2, P3, P4, P5) (*P* < .001), and the thickness of mandibular ramus was not completely equal from top to bottom at the 5 measure points (*P* < .001), with the thickest bone structure at point M4 (*P* < .001) and decreased at point M5 (*P*_M4vsM5_ < .001). The changes of thicknesses of the mandibular ramus are shown in Figure 7.

**Table 5:**
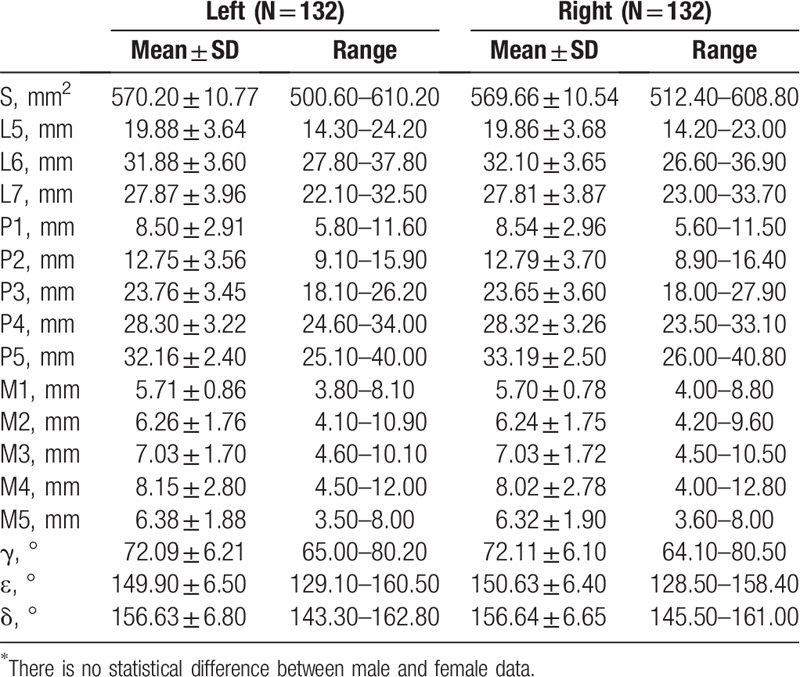
Width, height, and angle of the measurement points on mandibular ramus between 2 sides.

**Table 6:**
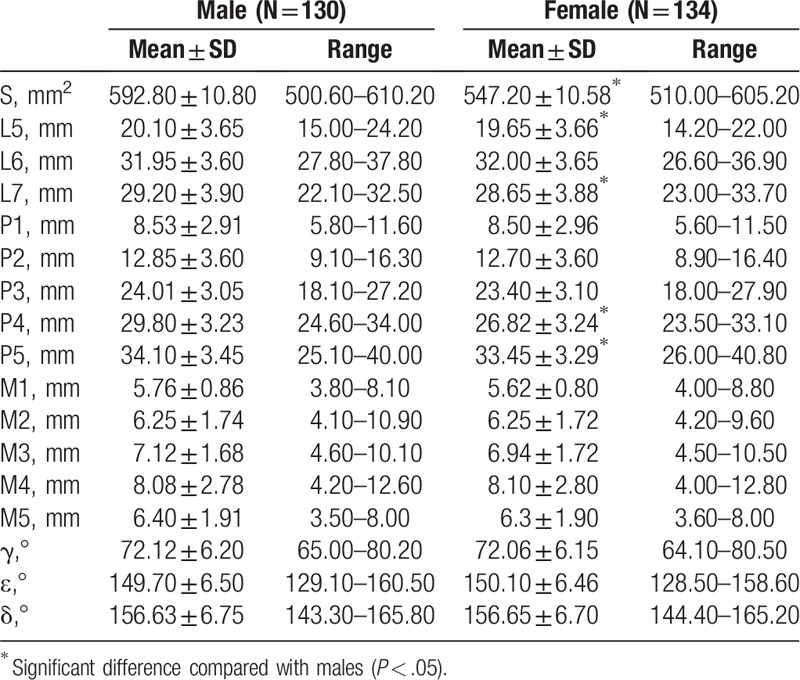
Width, height, and angle of the measurement points on mandibular ramus between 2 genders.

## 4 Discussion

TMJ has complex anatomical structures, which are involved in a series of physiological functions, such as speaking, chewing, swallowing, and so on. Certain TMJ diseases, including advanced TMJ arthropathy, tumors, comminuted fracture, may need removal of the original joint and reconstruction of the TMJ anatomical structure.^[1,8]^ The goal of the therapy is to restore the important physiological functions of the TMJ and improve the quality of life of the patients.^[9–12]^ Among the commonly used TMJ prosthesis, standard Biomet/Lorenz prosthesis (United States) is the only one that has been approved to be used in clinical practice in China.^[6]^ Standard Biomet artificial joint has 3 sizes (large, medium, and small) for clinical selection, but it is designed based on TMJ anatomical data of the Caucasians. The interracial morphological differences in skull structure might be associated with the developmental heterogeneity in cranial base morphology. Chang et al^[13]^ used thin-plate to compare the differences of the spline lateral cephalometric radiographs of the cranial base and the upper midface configuration between the European and the Asian, and demonstrated structural differences especially in horizontal compression and vertical expansion in the anterior portion of the cranial base in Asian. Besides, an anterior displacement of the TMJ and a relative forward position of the mandible were also noted.^[13]^ Therefore, the position of the screw, the size, and curvature of the prosthesis body are not entirely suitable for Chinese population.^[14]^ It is of great significance to measure and enrich the Chinese TMJ anatomical database for the research and development of Chinese standard TMJ prosthesis.

Standard TMJ prosthesis is composed of 2 parts that are fixed on the zygomatic arch and on the mandibular ramus, respectively. The stability of prosthesis is extremely critical in the process of artificial TMJ prosthesis replacement, which mainly depends on the fixed position of screws, the depth of fixation, and the degree and angle of the prosthesis and bone surface, etc. Previous studies have been limited to the measurement of the articular fossa and condylar process of TMJ.^[15,16]^ In our study, CT reconstruction image was used to measure the bony structure of TMJ, which has the advantages of high-quality imaging, free image cutting and accurate measurement, etc.^[17,18]^ On the basis of the analysis of a large number of samples, our study indicated that bone thickness was limited in the processus aboralis of zygomatic arch end joint, and corresponding screw length should be less than 5.82 ± 1.14 mm to avoid the screw entering into the cranial fossa to cause brain damage. The bone structure of the zygomatic arch was the thickest at the highest point of the lateral margin of the articular fossa among the 4 fixation points, with a rather small width. The existing screw fixing holes are designed in 2 rows and placed vertically in the zygomatic arch section of the standard TMJ prosthesis. Our measurements indicated that the zygomatic fossa at the lateral margin was narrow in width, and only 1 row of screw holes could be fixed according to the research results. The values of α and β were close to 180° on the medial side. In other words, the selected point of zygomatic arch approximately has the characteristic of collinearity above the skull, and a small amount of bone needed be grinded into a plane to fit the prosthesis during operation.

The bone contact surface of TMJ prosthesis was a flat plain. It was necessary to grind the bone to fit the prosthesis to the bone surface when placed. By measuring the length of BD, H1, and D5 in the articular fossa, the depth of the lateral landmarks from the articular fossa to the medial wall of the articular fossa, it was found that the anteroposterior diameter, average height, and transversal diameter of articular fossa were about 36.85 ± 4.65, 4.25 ± 1.89, and 19.72 ± 3.60 mm respectively, in Chinese population. It provided the data basis for the surgery selection of bone-grinding and autogenous bone graft in clinical practice.

As the Standard Biomet/Lorenz prosthesis was designed based on the TMJ morphology of the Caucasians, the application of such prosthesis in Chinese population might result in the risk of injury in inferior alveolar nerves and blood vessels, causing severe consequences, including numbness, pain, and loss of sensation in the dominant area.^[19,20]^ In this study, the measured mandibular foramen was located at 31.99 ± 3.62 mm in the direction of the extension of the angle, where the straight line was rearward in 72.10 ± 6.15° at the lowest point of the mandibular notch and orbitomeatal line. The safe fixation region of the mandibular ramus was determined by measuring the distance from the lower edge of the mandibular canal to the lower end of the mandibular ramus, and the region enclosed by mandibular canal, the posterior border of mandibular ramus, and line segments between them, which could be used to guide and design the number and quantity of screw holes. We measured the lengths from the projection of the mandibular canal to the posterior edge of the mandibular ramus, which were significantly increased from top to bottom, indicating the number of fixing screws could be properly increased to enhance the soundness of the prosthesis. Furthermore, the last 2 lengths in male were longer than those in female, which indicated that the screw fixation in this region is more flexible among male recipients and the gender factors should be considered during the prosthesis design. In addition, the lengths mentioned above corresponded to the safe distances from the mandibular canal to the posterior edge of the mandibular ramus. The measurements could provide guidance for the design of the number and fixation region of screw, while the differences in the measurements between 2 genders should also be noted. The thickness of the bone structure gradually increased from top to bottom, while slightly decreased at the fifth measure point, offering possible reference and detail for the choice of suitable screw type. Furthermore, the measurement of interior inclination angle and exterior inclination angle provided information on the design of the prosthesis to make it fit the bone surface better and improve the stability of the prosthesis.

The limitation is that our study is a single-center study and focuses on Chinese northeast population. On the contrary, there are officially 56 ethnic groups in China, including a majority group (ethnic Han) and 55 minority groups.^[21]^ There are no investigation focusing on the differences among regions and ethnic groups in China currently. A majority of the selected volunteers in our study are Chinese Hans, and there might be differences in the measurements among the ethnic groups and regions in China. Therefore, the measurements data obtained from the hospital are suitable for the ethnic Han population in the northeast part of China. In the further study, the anatomical data differences between different regions and different ethnic groups will be explored through multicenter, large-scale investigations.

Individual variation should never be ignored when planning and performing TMJ replacement. Our study provided the range of measurements of various landmarks, which could contribute to the clarification of the most reasonable measurements for tailoring standard TMJ prosthesis for Chinese northeast population and the identification of the reference range for typing. Data mining methods such as cluster analysis could be used to group the TMJ samples into clusters to help each individual patient find the best-fitting prosthesis.^[6]^

## 5 Conclusion

In this study, CT reconstruction technique was used to measure a large number of skull samples on the basis of relevant research.^[6,11,15]^ Detailed analysis and in-depth survey of fixed region of prosthetic screw were conducted by measuring the zygomatic arch width, thickness, length, screw fixation region, mandibular ramus thickness and length at the landmarks, etc. In addition, human variability should be considered in clinical practice. The study described the fixation region that was available for the TMJ prosthesis and clarified the safe region for fixation, which enriched the anatomy database of TMJ in Chinese northeast population, and provided data support for the design of standard TMJ prosthesis for Chinese people.

## Acknowledgment

The authors thank the patients for their participation in this study.

## Author contributions

**Investigation:** Yingying Han, Zhuan Zhong, Chunyang Kang.

**Methodology:** Yingying Han, Zhuan Zhong, Chunyang Kang.

**Project administration:** Zhuan Zhong.

**Resources:** Jialiang Sun.

**Software:** Chunyang Kang.

**Validation:** Zhentao Yu.

**Visualization:** Zhentao Yu, Chunyang Kang.

**Writing – original draft:** Jialiang Sun, Zhentao Yu.
